# Rice bran oil ameliorates inflammatory responses by enhancing mitochondrial respiration in murine macrophages

**DOI:** 10.1371/journal.pone.0222857

**Published:** 2019-10-11

**Authors:** Sojung Lee, Seungmin Yu, Hye Jeong Park, Jiyeon Jung, Gwang-woong Go, Wooki Kim

**Affiliations:** 1 Department of Food Science and Biotechnology, Graduate School of Biotechnology, Kyung Hee University, Yongin-si, Gyeonggi-do, Korea; 2 Department of Food and Nutrition, Hanyang University, Seongdong-gu, Seoul, Korea; University of PECS Medical School, HUNGARY

## Abstract

Previous studies have revealed the anti-inflammatory properties of rice bran oil (RBO), but the detailed mechanisms are poorly understood. Recent studies on the molecular/cellular anti-inflammatory mechanisms of dietary components have demonstrated that mitochondrial respiration plays a key role in macrophage functioning. Since dietary lipids are major substrates for mitochondrial respiration through β-oxidation, the current study examined whether RBO regulates inflammatory responses by modulating mitochondrial energy metabolism. Palm oil (PO), enriched with palmitic acid which are known to be effectively taken up by cells and used for oxidative phosphorylation, served as a positive control. In the *in vitro* model of LPS-stimulated RAW 264.7 murine cells, the levels of pro-inflammatory cytokines (IL-6 and TNF-α) in the culture supernatant were significantly reduced by RBO treatment. In contrast, secretion of the anti-inflammatory cytokine IL-10 was upregulated by RBO. Transcription of genes encoding inflammatory mediator molecules (COX-2 and iNOS) and expression of activation markers (CD80, CD86, and MHC-II) in LPS-stimulated RAW 264.7 cells were suppressed by RBO. Mitochondrial respiration (as assessed by an extracellular flux analyzer) increased upon RBO treatment, as the basal respiration, maximal respiration, ATP production, and spare respiratory capacity were upregulated. In an *in vivo* study, C57BL/6 mice were fed a negative control diet containing corn oil (CO), PO, or RBO for 4 weeks, and bone marrow-derived macrophages (BMDM) were isolated from their tibias and femurs. In pro-inflammatory M1-polarized BMDM (M1-BMDM), the RBO-induced suppression of IL-6 and TNF-α was recapitulated *in vivo*. Mitochondrial respiration in M1-BMDM also increased following the RBO intervention and the PO control treatment as compared to CO fed negative control. Overall, the current study for the first time demonstrates that RBO regulates inflammatory responses in murine macrophages by upregulating mitochondrial respiration. Further clinical studies are required to validate the animal study.

## Introduction

It is well established that eukaryotes utilize glucose as a major cellular energy substrate through glycolysis to the intermediate product of pyruvate. Under aerobic conditions, pyruvate is oxidized to acetyl-CoA, which is further oxidized through the Krebs cycle, producing proton-bearing molecules such as NADH and FADH_2_ [1]. These molecules contribute to the proton gradient through mitochondrial membranes, thereby producing ATP at the expense of oxygen in the process termed as mitochondrial respiration. On the other hand, under anaerobic conditions, due to the lack of oxygen molecules, the intermediate pyruvate molecules undergo lactate fermentation rather than mitochondrial respiration, thereby producing protons in the extracellular matrix. However, Dr. Warburg, a Nobel Laureate, demonstrated that normal cells utilize mitochondrial respiration, while “urgent” cells such as cancer cells and inflammatory cells prefer lactate fermentation, even when oxygen is abundant [2]. It should be highlighted that, in experimental approaches to elucidate these phenomena, state-of-the-art technology was developed to finely assess oxygen consumption (pmoles) and proton production (mpH) in the micromilieu of cell cultures [3].

At the onset of inflammation, macrophages play a major role in phagocytosis of microbes and subsequent secretion of cytokines and chemokines [4]. Macrophages also express antigen-presenting molecules and co-stimulatory proteins on the cell surface for further engagement in adaptive immune responses [5]. However, under certain conditions, macrophages have been shown to contribute to the prevention and/or resolution of inflammation. Consequently, macrophages have been subcategorized into at least two categories: M1 classically activated inflammatory macrophages and M2 alternatively activated anti-inflammatory macrophages [6,7]. Recently, it was reported that mitochondrial respiration is important for macrophage polarization and function [1,8], and that lysosomal lipolysis in macrophages is essential for M2 polarization [9]. It is notable that cellular lipid metabolism is regulated by dietary oils. Indeed, short- and medium-chain fatty acids were shown to be effective substrates for β-oxidation because they have a higher affinity for the aqueous cytoplasm than conventional long-chain fatty acids. The subsequent increase in acetyl-CoA upregulates mitochondrial respiration [10].

Chronic inflammation is one of the health risks and preventative trials using natural products as dietary supplements have been approached [11–14]. In this regard, the anti-inflammatory effects of pinocembrin, a flavanone, was shown to be augmented by acylation with fatty acids [15], indicating the chances of development of safer drugs from natural resources. While multiple cellular signaling pathways involved in inflammatory responses revealed, cyclooxygenase (COX) was targeted to one of key regulators in dietary approaches [16,17]. Rice bran oil (RBO) is an edible oil extracted from the hard, outer layer of rice and is well known for its health-beneficial components, including γ-oryzanol and tocopherols [18]. In this regard, the anti-inflammatory effects of γ-oryzanol in RBO was reported in rat peritoneal macrophage *in vivo* [19]. Furthermore, previous studies on the health-beneficial effects of RBO, including anti-obesity and anti-diabetic effects, were reviewed in cellular, animal, and clinical models [20,21], yet the detailed cellular mechanisms have not been fully elucidated. Therefore, the current study examined the molecular mechanisms of RBO activity in inflammatory model systems. It was hypothesized that RBO suppresses macrophage functions by upregulating mitochondrial respiration in inflammatory states. Positive control palm oil (PO), largely consisted of palmitic acid (C16:0), was previously shown to be effective in β-oxidation and increased mitochondrial respiration. In contrast, corn oil (CO), one of the most consumed edible oil worldwide [22], served as a negative control.

## Methods

### Cell culture and treatment

The murine macrophage cell line RAW 264.7 was purchased from the Korea Cell Line Bank (Seoul, Korea). The cells were cultured in Dulbecco’s modified Eagle’s medium (DMEM) supplemented with 10% fetal bovine serum (FBS, Thermo Fisher Scientific, Waltham, MA, USA) and 1% penicillin/streptomycin (Thermo Fisher Scientific) at 37°C in an atmosphere of 5% CO_2_ and were cultured between five to eight passages for the experiments. Commercially available RBO and PO were purchased from Serim Hyunmi (Jungeup-si, Korea) and JCY (Seongnam-si, Korea), respectively. For *in vitro* treatments, the oils were dissolved in DMSO, for which the final concentration in the culture medium was kept under 0.01% (v/v). Following oil treatment for 24 hrs, the cells were stimulated with LPS at a concentration of 500 ng/mL, and the culture supernatant was harvested by centrifugation at 500 x *g* for cytokine quantification. The cell pellets were used for mRNA assays.

### Animal study and M1-BMDM polarization

C57BL/6 mouse is a well-defined animal model for the dietary intervention and immunological assessments. Male mice at 4–6 wks were purchased from Raon Bio (Yongin-si, Korea) and acclimated with a rodent chow diet for 1 wk. The composition of each experimental diet was modified from that of the AIN-76A semi-purified diet, as shown in Table 1. Mice were randomly divided into three groups (n = 7–8) and fed the respective diets for 4 wks *ad libitum* under a climate-controlled 12h/12h dark/light cycle. Following the dietary intervention, the mice were euthanized by CO_2_ inhalation, and bone marrow cells were isolated from their tibias and femurs in an aseptic environment. Dietary intervention studies were conducted in accordance with guidelines approved by the Kyung Hee University IACUC (specific approval number for the current study: KHUASP(GC)-18-017). For induction of bone marrow-derived macrophages (BMDM), cells were cultured in Iscove’s modified Dulbecco’s medium (IMDM, Thermo Fisher Scientific) supplemented with 10% FBS and 10 ng/mL macrophage colony-stimulating factor (M-CSF, Thermo Fisher Scientific) for 7 days. Following BMDM induction, the cells were further polarized to inflammatory M1 macrophages (M1-BMDM) by culturing in IMDM supplemented with 10% FBS, 100 ng/mL LPS, and 50 ng/mL IFN-γ [23].

**Table 1:** Experimental diet composition.

Ingredients	Composition (g/100g)		
	CO^1)^	PO	RBO
Casein	200	200	200
DL-methionine	3	3	3
Sucrose	499.99	499.99	499.99
Corn starch	150	150	150
Corn oil	50	0	0
Palm oil	0	50	0
Rice bran oil	0	0	50
Cellulose	50	50	50
Mineral mix, AIN-76	35	35	35
Vitamin mix, AIN-76A	10	10	10
Choline bitartrate	2	2	2
Ethoxyquin, antioxidant	0.01	0.01	0.01

^1)^The AIN-76A semi-purified rodent diet was modified with different edible oils. (CO, corn oil; PO, palm oil; RBO, rice bran oil)

### Analysis of fatty acid composition by gas chromatography (GC)

Total lipid was extracted from vegetable oils by Folch method (1957). Saponification was completed using a 0.5 N potassium hydroxide (Sigma-Aldrich), followed by methylation using 13–15% boron trifluoride (Sigma-Aldrich) to yield fatty acid methyl ester (FAME) (Smith et al., 2002). FAMEs were dissolved using hexane and were dehydrogenated over anhydrous Na_2_SO_4_. Fatty acid profiles were identified by gas chromatography. FAME sample was injected into a gas chromatograph with FID (Agilent 7890N, Santa Clara, CA, USA) fitted with a capillary column (Omegawax; 30 m x 0.25 mm; id,0.25 μm, Sigma-Aldrich). The initial temperature was set at 100°C, followed by a gradually increasing rate of 2°C/min, until reaching a final temperature of 220°C, which was maintained for 15 min. The injector and detector temperatures were set at 250°C and 260°C, with a split ratio of 20. Fatty acid was identified by comparing with the retention times of 35 components FAME mixture (CRM47885, Sigma-Aldrich). Identified peaks were presented as peak area per total area of detected fatty acids (g/100 g oil).

### Cytokine and mRNA assessments

The culture supernatants were stored at -80°C until used. For analysis of cytokines in the culture media, a BD^TM^ Cytometric Bead Array Mouse Inflammation Kit (BD Biosciences, San Jose, CA, USA) was used in accordance with the manufacturer’s guidelines. Briefly, the supernatants were incubated with a mixture of capture beads specific to IL-6, IL-10, MCP-1, IFN-γ, TNF-α, and IL-12p70. Following incubation, excess beads were removed by centrifugation, and a PE-conjugated detection antibody was applied. The cytokine-bead conjugates were analyzed on a BD Accuri^TM^ C6 flow cytometer (BD Biosciences), with the FL1 channel for discrimination of the beads and the FL2 channel for quantification of the captured cytokines. Appropriate standard cytokines and analytic software were provided in the assay kit.

Total RNA was isolated from cells with an MG Total RNA Extraction Kit (MG Med, Seoul, Korea) according to the manufacturer’s instructions. The RNA concentration and purity were assessed on a Nanodrop 2000 spectrophotometer (Thermo Scientific). Inflammatory gene transcription was measured by quantitative reverse-transcriptase PCR (qRT-PCR) with MG One-step RT-PCR MasterMix (SYBR Green) (MG Med) and a CFX Connect^TM^ Real-Time System (Bio-Rad, Hercules, CA, USA) under the following conditions: 42°C for 10 min, 95°C for 10 min, followed by 39 amplification cycles at 95°C for 5 s and 55°C for 30 s. Following qRT-PCR reactions using the primers shown in Table 2, the 2^-ΔCt^ method was used to calculate the relative mRNA expression level of the target gene compared to the internal control gene (GAPDH), through the use of Bio-Rad CFX manager software. Specifically, ΔCt was calculated as Ct_target gene_−Ct_GAPDH_, where Ct represents the amplification detection cycle number of an arbitrary threshold.

**Table 2:** Primer sequences for qRT-PCR quantification of inflammatory mediators.

Gene	Sequence	
mGAPDH	Forward	5’-CACTCACGGCAAATTCAACGGC-3’
	Reverse	5’-CCTTGGCAGCACCAGTGGATGCAGG-3’
mCOX-2	Forward	5’-TCTCAGCACCCACCCGCTCA-3’
	Reverse	5´-TTTGACCTCAGCGCTCAGTTG-3´
miNOS	Forward	5’-CCCTTCCGAAGTTTCTGGCAGCAGC-3’
	Reverse	5´-GGCTGTCAGAGCCTCGTGGCTTTGG-3´

### Quantification of macrophage surface-expressed activation markers

The surface expression of activation markers on macrophages was analyzed by flow cytometric analysis. The aforementioned LPS-stimulated cells were washed with cold PBS and treated with anti-mouse CD16/CD32 mAbs at 4°C to block non-specific antibody binding in the Fc region. Cells were subsequently stained with fluorescence-conjugated mAbs (FITC-conjugated anti-mouse CD80, PE-conjugated anti-mouse CD86, and APC-conjugated anti-mouse MHC class II) for 10–15 min at 4°C. After the cells were stained with the specific antibodies, excess cold PBS was used to wash the cells, and the cells were centrifuged at 300 x *g* for 5 min. A flow cytometer (BD Accuri^™^ C6) was used to analyze the expression of the activation markers on the macrophages, and the mean fluorescence intensities (MFIs) were quantified with BD Accuri^TM^ C6 software.

### Extracellular flux analysis

RAW 264.7 macrophages or M1-BMDM were plated at 1×10^4^ cells/well in Seahorse^TM^ XFp assay plates (Agilent Technologies, Santa Clara, CA, USA). The RAW 264.7 cells were further treated with DMSO-dissolved oils for 24 hrs at 37°C in a 5% CO_2_ incubator, followed by LPS (500 ng/mL) stimulation for another 24 hrs. The LPS-stimulated RAW 264.7 cells or M1-BMDM were supplemented with DMEM containing 4,500 mg/L D-glucose, L-glutamine, and sodium pyruvate at pH 7.4, and were incubated at 37°C in a non-CO_2_ incubator for 1 hr. The mitochondrial inhibitors oligomycin (1.0 μM), FCCP (1.0 μM), and rotenone/antimycin A (0.5 μM) were added to the cells sequentially, in accordance with the manufacturer’s recommendations. The oxygen consumption rate, an indicator of oxidative phosphorylation, was automatically monitored by the Seahorse^TM^ XFp analyzer and software (Agilent Technologies), enabling quantification of basal respiration, maximal respiration, ATP production, and spare respiration capacity.

### Statistics

The statistical significance of differences among treatments was determined by one-way ANOVA, followed by Tukey’s post-hoc multiple comparisons. GraphPad Prism 5 software (La Jolla, CA, USA) was used. Data are presented as mean with SEM, and p<0.05 was considered significant.

## Results

### Fatty acid composition of edible oils

Commercial oils used in the current study, i.e., CO, PO, and RBO, were identified for their fatty acid composition as shown in Table 3. Briefly, CO was identified to be highly enriched in linoleic acid (C18:2n6, 49.75%) followed by oleic acid (18:1n9, 36.79%) as previously reported. The fatty acid composition in PO exhibited 39.89% palmitic acid (C16:0), indicating that PO contains two-carbon-shorter fatty acids as compared to 18-carboned long chain fatty acids. With regard to palmitic acid content, RBO (13.98%) was identified to be in the middle of CO and PO, where oleic acid (18:1n9) composed 49.65% of RBO.

**Table 3:** Fatty acid composition in the vegetable oils (g /100 g total fatty acids).

Fatty acid	CO	PO	RBO
C12:0	nd	0.13±0.10	nd
C14:0	nd	0.98±0.01	nd
C16:0	10.36±0.17	39.89±0.08	13.98±0.05
C18:0	1.80±0.02	4.25±0.03	1.29±0.01
C18:1n9	36.79±0.04	47.15±0.11	49.65±0.05
C18:2n6	49.75±0.17	7.43±0.00	33.45±0.00
C18:3n3	0.93±0.01	Nd	0.67±0.01
C20:0	0.38±0.01	0.17±0.14	0.52±0.00
C20:4n6	nd	nd	nd^1)^
C20:5n3	nd	nd	nd
C22:6n3	nd	nd	nd
SFA^2)^	12.53±0.17	41.17±0.133	14.49±0.05
MUFA^3)^	36.79±0.04	47.15±0.11	49.65±0.05
PUFA^4)^	50.68±0.17	7.43±0.00	34.12±0.00

^1)^nd, not detected

^2)^SFA, saturated fatty acid: the sum of C12:0, C14:0, C16:0, C18:0, and C20:0

^3)^MUFA, mono-unsaturated fatty acid: C18:1n9

^4)^PUFA, poly-unsaturated fatty acid: the sum of C18:2n6, C18:3n3, C20:4n6, C20:5n3, and C22:6n3

### Suppressed secretion of pro-inflammatory cytokines by RBO

**Fig 1:**
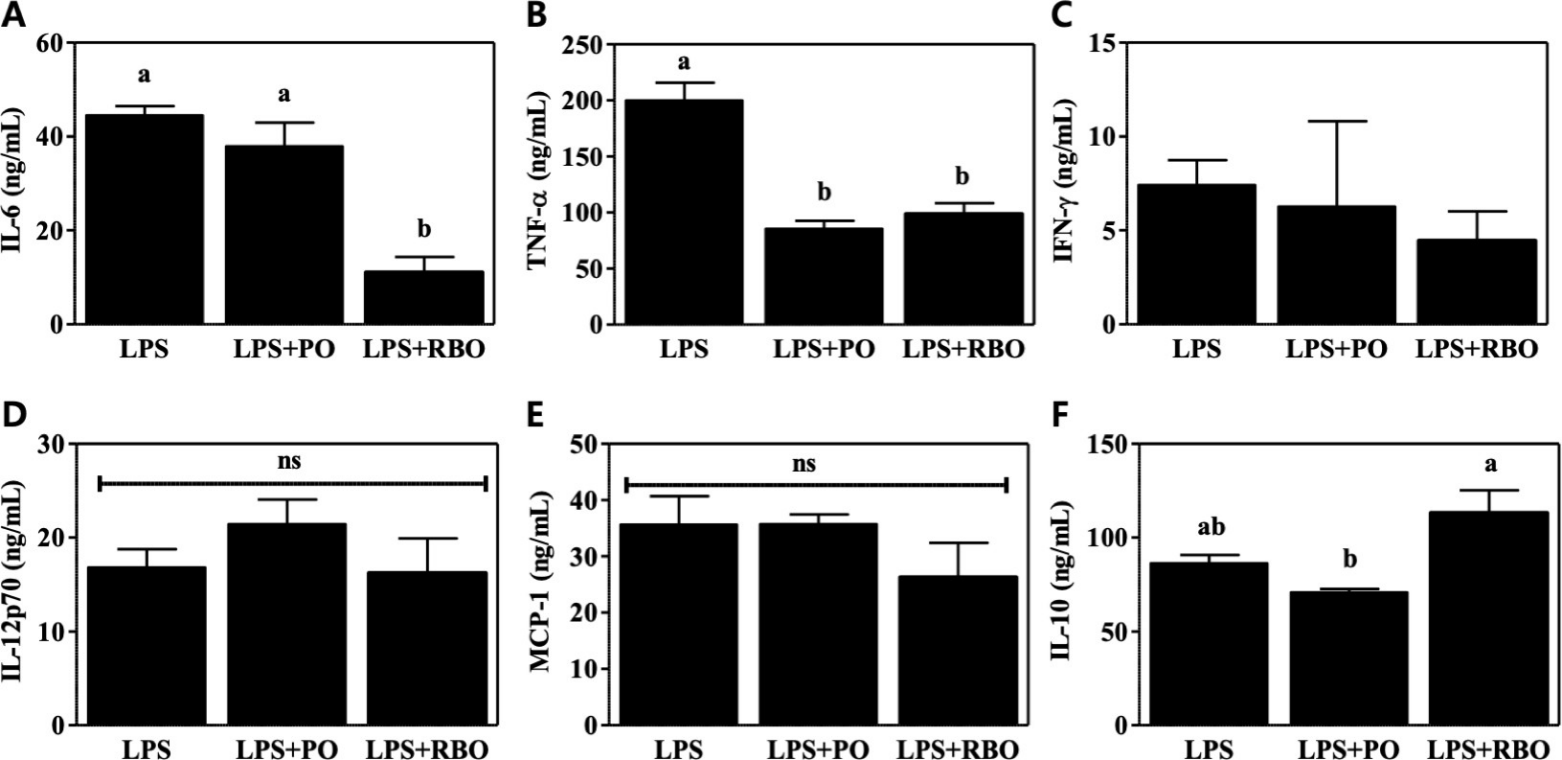
Cytokine production by LPS-stimulated RAW 264.7 macrophages, as assessed with a multiple-cytokine bead array. Data are presented as mean±SEM (n = 5). Values with different letters are statistically significant at p<0.05 within the panel. (PO, palm oil; RBO, rice bran oil).

Inflammatory responses were induced in RAW 264.7 cells by addition of LPS to the medium. Following supernatant harvest, multiple cytokines were quantified. LPS induced the production of IL-6, a pro-inflammatory cytokine (44.45±1.999 ng/mL), but this induction was significantly suppressed by the addition of RBO (11.06±3.245 ng/mL, P<0.05), whereas PO, which served as a control, did not affect the IL-6 quantity (37.92±5.014 ng/mL) (Fig 1A). TNF-α secretion was downregulated by both the RBO treatment (98.57±9.810 ng/mL, P<0.05) and the PO treatment (85.36±7.138, P<0.05) compared to the LPS control treatment (199.9±16.10 ng/mL) (Fig 1B). Other pro-inflammatory cytokines (IFN-γ, IL12p70, and MCP-1) were not affected by the oil treatments (P>0.05, Fig 1C–1E). Interestingly, production of anti-inflammatory cytokine IL-10 was greater in the RBO group (113.2±12.03 ng/mL, P<0.05) than in the PO control group (70.83±1.815 ng/mL) (Fig 1F).

**Fig 2:**
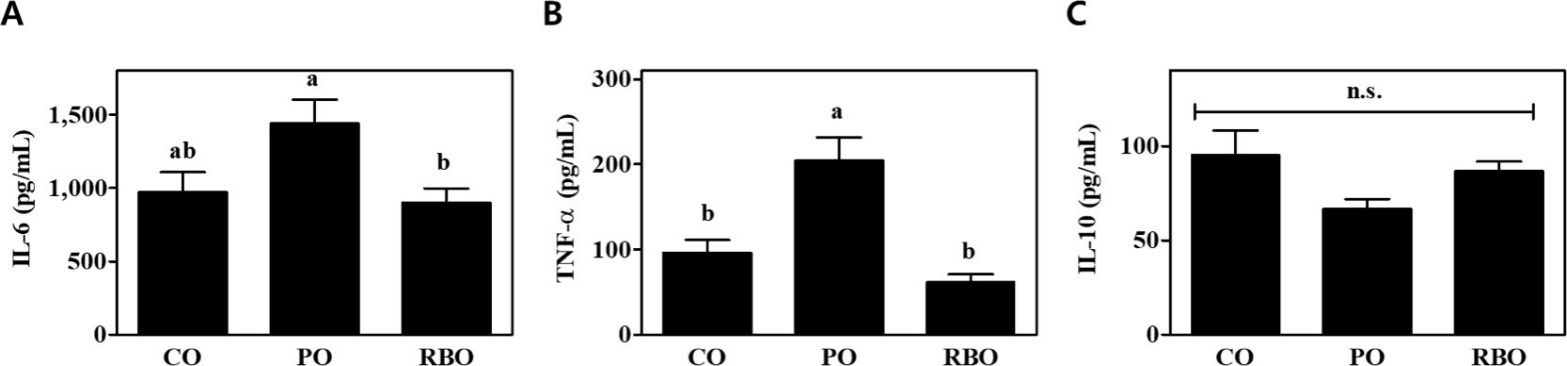
Cytokine production by M1-polarized BMDM following dietary intervention with an AIN-76A-based diet containing either CO, PO, or RBO for 4 wks. Data are presented as mean±SEM (n = 7–8). Values with different letters are statistically significant at p<0.05 within the panel. (CO, corn oil; PO, palm oil; RBO, rice bran oil).

Next, the anti-inflammatory effects of RBO were investigated *in vivo*. C57BL/6 male mice at the age of 4–6 weeks were fed a semi-purified AIN-76A diet including 5% of either CO, PO, or RBO as the dietary lipid source. Following 4 weeks of feeding *ad libitum*, there was no difference in body weight gain among the dietary groups (Table 4). Bone marrow cells were isolated following the dietary intervention, and M1-BMDM were induced by a well-established protocol [23]. The secretion of IL-6 into the culture medium was highest in the PO-fed group (1,439±163.3 pg/mL), but was reduced to 901.8±94.29 pg/mL by RBO feeding (P<0.05) (Fig 2A). The secretion of TNF-α also showed that PO increased the inflammatory response (207.1±27.49 pg/mL) compared to the CO control (96.14±15.34 pg/mL, P<0.05), while the RBO intervention (61.74±9.025 pg/mL) yielded a value comparable to that of the CO control group (Fig 2B). The production of IL-10, an anti-inflammatory cytokine, was detectable in the M1-BMDM supernatant, but there was no significant difference among the dietary groups (CO 95.11±13.31 vs. PO 66.51±5.386 vs. RBO 86.57±5.321 pg/mL) (Fig 2C).

**Table 4:** Body weight changes following dietary intervention.

	CO^1)^	PO	RBO
Initial weight (g)	15.55 ± 0.1611	15.53 ± 0.1602	15.56 ± 0.1842
Final weight (g)	26.69 ± 0.6891	27.47 ± 0.7425	27.24 ± 0.7820

^1)^The AIN-76A semi-purified rodent diet was modified with different edible oils. (CO, corn oil; PO, palm oil; RBO, rice bran oil)

### Downregulation of gene transcription for inflammation mediators

**Fig 3:**
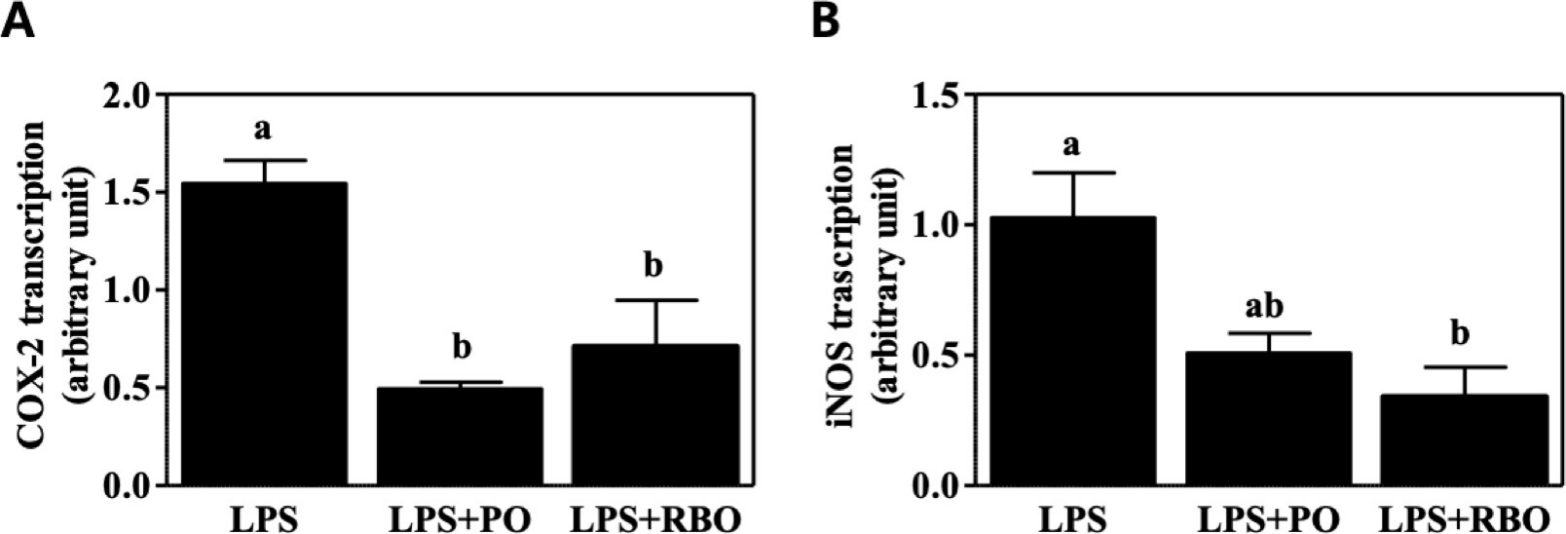
Transcription of COX-2 and iNOS, inflammatory mediator genes, as assessed by qRT-PCR in LPS-stimulated RAW 264.7 macrophages. Data are presented as mean±SEM (n = 3). Values with different letters are statistically significant at p<0.05 within the panel. (PO, palm oil; RBO, rice bran oil).

Transcription of genes encoding inflammatory mediators, i.e., COX-2 and iNOS, was quantified by qRT-PCR. The PO control and RBO suppressed COX-2 transcription by 68.03% and 54.03%, respectively, compared to the LPS control (Fig 3A). In contrast, transcription of iNOS was significantly reduced by RBO (66.84%, P<0.05) but not by PO (P>0.05) compared to the LPS control (Fig 3B).

### Reduced expression of macrophage surface markers

**Fig 4:**
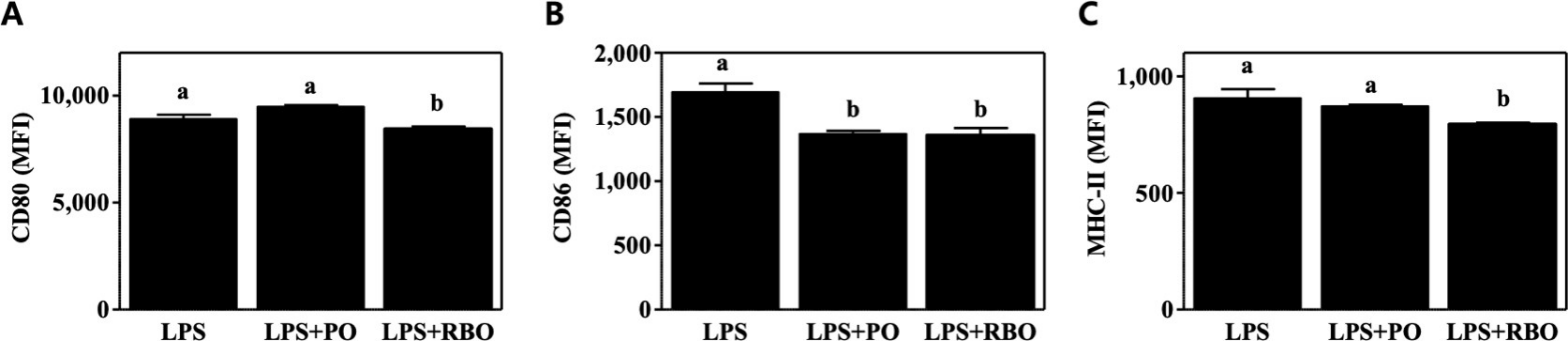
Expression of activation markers on the surface of LPS-stimulated RAW 264.7 macrophages. Cells were stained with fluorescence-conjugated mAbs specific to (A) CD80, (B) CD86, or (C) MHC-II, and flow cytometric quantification was performed. Data are presented as mean±SEM (n = 7–8) of arbitrary units of mean fluorescence intensity (MFI). Values with different letters are statistically significant at p<0.05 within the panel. (PO, palm oil; RBO, rice bran oil).

The expression of co-stimulatory (CD80 and CD86) and antigen-presenting (MHC-II) molecules was quantified through incubation of LPS-stimulated cells with fluorescence-conjugated antibodies, followed by flow cytometric analysis. In terms of MFI, the expression of CD80 was slightly but significantly lower in the RBO treatment group (8,438±119.4, P<0.05) than in the LPS control group (8,891±209.8) (Fig 4A). Both PO and RBO suppressed the expression of CD86, with comparable MFIs (1,368±24.37 and 1,356±58.69, respectively) (Fig 4B). RBO also downregulated the expression of MHC-II (793.4±7.07, P<0.05) vs. the LPS control (904.8±40.17) (Fig 4C).

### Enhancement of mitochondrial respiration

**Fig 5:**
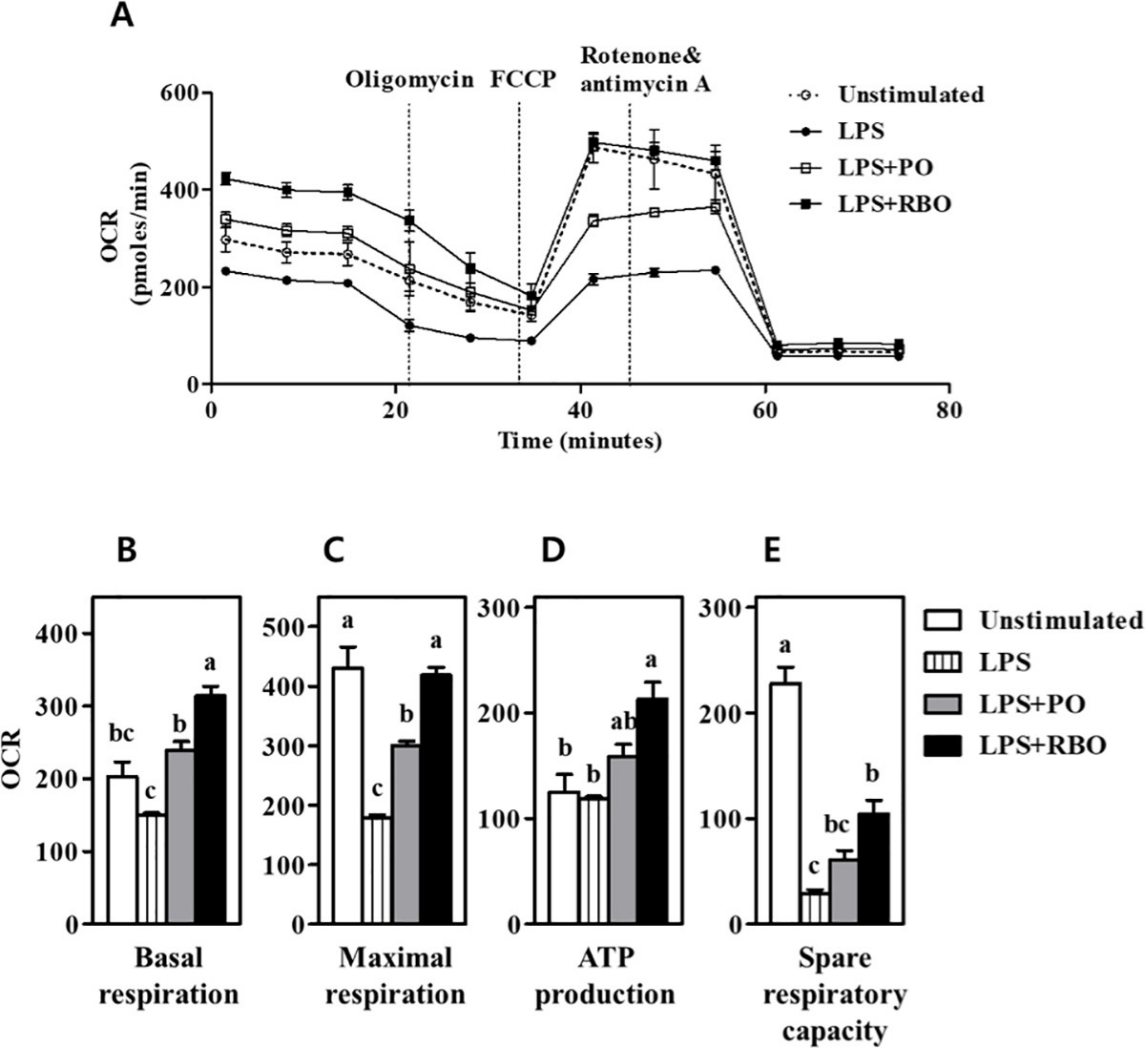
Quantification of mitochondrial respiration, as assessed by the oxygen consumption rate (OCR) determined with a SeahorseTM extracellular flux analyzer in RAW 264.7 macrophages. (A) Real-time OCR quantification of activated and/or oil-treated cells, with appropriate drugs to control mitochondrial respiration, resulting in analysis of (B) basal respiration, (C) maximal respiration, (D) ATP production, and (E) spare respiratory capacity. Values with different letters are statistically significant at p<0.05 within the panel. (PO, palm oil; RBO, rice bran oil).

Mitochondrial respiration, as determined by oxygen consumption rate (OCR), was quantified with a Seahorse^TM^ cellular flux analyzer. Unstimulated RAW 264.7 cells exhibited typical responses to cellular energy capacity-determining drugs, i.e., oligomycin, FCCP, and rotenone/antimycin A (Fig 5A, open circles). LPS-induced inflammatory stimulation dramatically reduced the OCR (Fig 5A, closed circles), while this effect was reversed by addition of PO (Fig 5A, open squares). Of interest, RBO treatment increased the OCR to the level of the unstimulated control group (Fig 5A, closed squares). The aforementioned drug treatments allowed separation of the cellular oxygen consumption capacity into basal respiration, maximal respiration, ATP production, and spare respiratory capacity [24]. In detail, the basal respiration of the cells stimulated with LPS (150.6±2.764 pmoles/min) did not differ from that of the unstimulated control (202.7±20.50 pmoles/min). However, basal respiration was significantly greater in the PO-treated group (314.1±13.15 pmoles/min, P<0.05) than in the LPS-stimulated control group. RBO (314.1±13.15 pmoles/min) further increased the basal respiration of the cells to a level exceeding that of the unstimulated control (P<0.05) (Fig 5B). The maximal respiration was 430.7±35.54 pmoles/min in unstimulated cells) and was reduced to 179.6±4.089 pmoles/min by LPS stimulation (P<0.05), while this effect was partially reversed by PO (300.5±7.165 pmoles/min) and fully reversed by RBO (418.6±13.16 pmoles/min) treatment (Fig 5C). Cellular oxygen consumption for ATP production was determined and was found not to be affected by LPS stimulation or PO treatment (unstimulated 125.3±16.70 vs. LPS 118.7±.440 vs. PO 158.7±11.72 pmoles/min) (P>0.05). However, the spare respiratory capacity was dramatically reduced by LPS treatment (29.04±3.777 pmoles/min) compared to the unstimulated control (228.0±15.56 pmoles/min, P<0.05). The spare respiratory capacity of PO-treated cells (60.95±8.821 pmoles/min) did not differ significantly from that of the LPS control, while that of the RBO group (104.05±12.55 pmoles/min) was significantly greater than that of the LPS-stimulated control (P<0.05).

**Fig 6:**
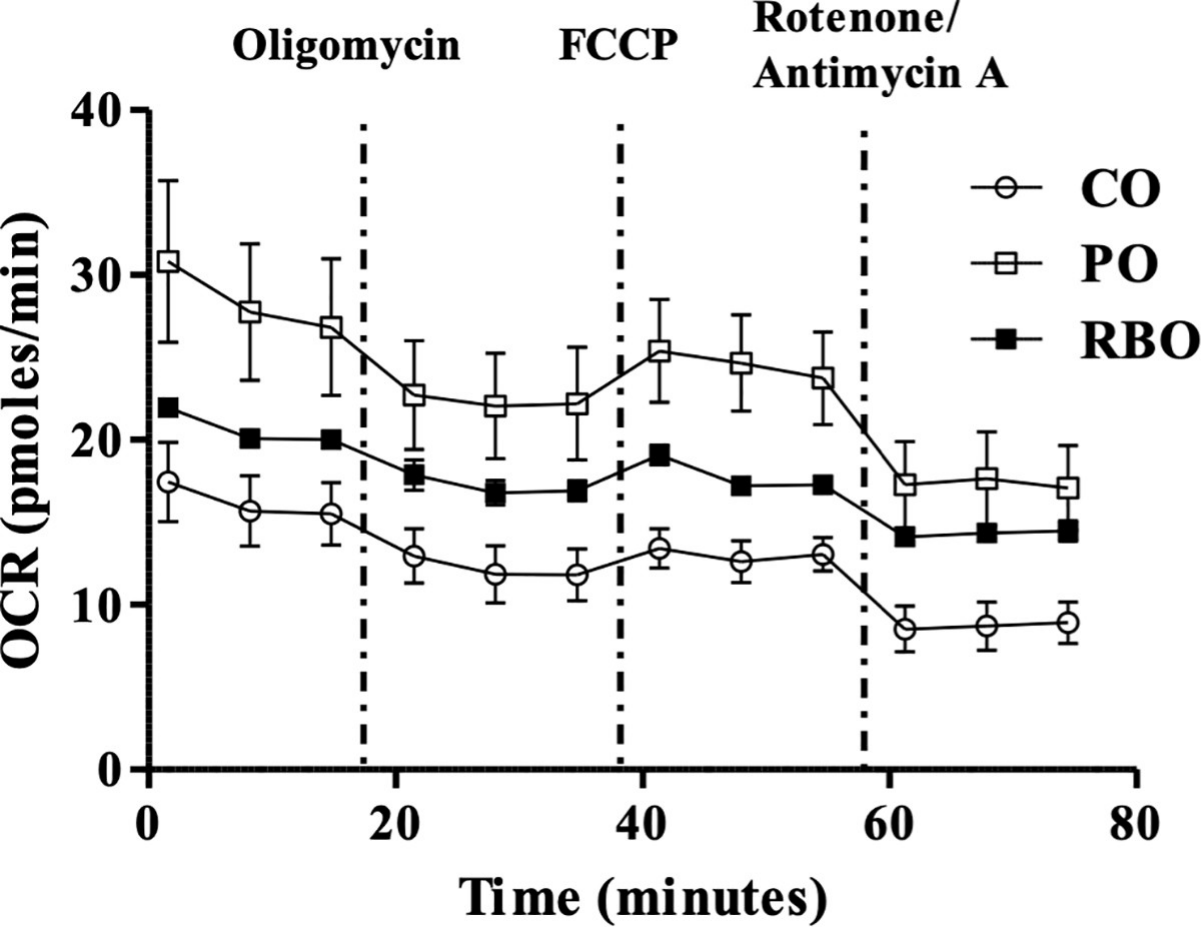
Real-time OCR quantification of M1-induced BMDM isolated from experimental diet-fed mice. Appropriate drugs were used to control mitochondrial respiration.

As an experiment to recapitulate the RBO-induced increase in mitochondrial respiration in a relevant animal model, M1-BMDM from the aforementioned diet-fed mice were analyzed. The OCR was greater in the PO (Fig 6, open squares) and RBO (closed squares) groups than in the CO-treated group (open dots).

## Discussion

The canonical role of macrophages (later termed as classically activated M1 macrophages) in inflammatory responses is production of pro-inflammatory cytokines via well-known signaling pathways, including the TLR4-MyD88-dependent NF-kB axis [6,25]. However, a number of studies have described alternatively activated macrophages generated in a process called M2 polarization. M2 macrophages are known to help resolve inflammation and repair tissues [7]. Though there has been much interest in the M1 vs. M2 polarization of macrophages, the determinant mechanisms are not fully understood.

With respect to the prevention and/or treatment of chronic inflammation, dietary compounds are receiving attention due to their safety and lack of adverse effects [26]. Among various dietary approaches, treatment with the edible oil RBO was investigated for its anti-inflammatory effects in the current study. Dietary lipids serve as major energy substrates to cells through β-oxidation and subsequent mitochondrial respiration [27]. Of interest, recent advances in macrophage studies have revealed that the cellular energy production pathway, i.e., glycolysis vs. oxidative phosphorylation, is closely related to M1 vs. M2 polarization in macrophages [1,28].

To test whether RBO suppresses inflammatory responses, LPS-stimulated RAW 264.7 cells, which are widely used as an inflammatory model system [29], were used. As shown in Fig 1A and 1B, secretion of the inflammatory cytokines IL-6 and TNF-α was significantly suppressed by RBO treatment, while secretion of the anti-inflammatory cytokine IL-10 was reciprocally upregulated (Fig 1F). In this regard, Islam et al. also reported that cycloartenyl ferulate extracted from rice bran downregulated the transcription of iNOS mRNA in an NF-kB-dependent manner in LPS-stimulated RAW 264.7 macrophages [30]. In contrast, Sierra et al. reported that RBO enhanced the production of Th1-prone cytokines (i.e., IL-2 and TNF-α) and the proliferation of B cells in splenocytes compared to high-oleic sunflower oil [31]. These conflicting observations may have resulted from the different cellular models. In addition, Yadav et al. reported that RBO ameliorated adjuvant-induced arthritis in rats [32], but the detailed cell types responsible for the RBO-induced suppression of inflammation were not clearly described. Therefore, in this study, BMDM were isolated following a 4-wk experimental dietary intervention, and the cells were further polarized to M1 cells by well-accepted *ex vivo* polarization methods [3]. The results recapitulated the *in vitro* observations, as IL-6 and TNF-α production were lower in the RBO group than in the PO group (Fig 2A and 2B). The amelioration of LPS-activated RAW 264.7 cells by RBO was further evidenced by quantification of inflammatory mediator mRNA levels (COX-2 and iNOS) (Fig 3) and activation markers on the cellular surface (CD80, CD86, and MHC-II) (Fig 4).

Following the observation of RBO-induced anti-inflammatory effects in macrophages, the cellular mechanisms were studied both *in vitro* and *ex vivo*. Cells treated with RBO followed by LPS exhibited an increased OCR curve compared to cells treated only with LPS (Fig 5A), indicating that RBO upregulates mitochondrial respiration. PO, largely composed of palmitic acid (C16:0) [33], served as a positive control, since many studies have demonstrated that shorter fatty acids are more susceptible than long-chain fatty acids to carnitine-independent cellular uptake and subsequent β-oxidation and mitochondrial respiration [34,35]. In the current study, the PO control successfully upregulated the OCR (Fig 5A), and the same phenomenon was observed in M1-polarized BMDM cells following a 4-wk dietary intervention (Fig 6). These data clearly supported the hypothesis that RBO, as well as the positive control PO, upregulates mitochondrial respiration in macrophages.

As shown in Fig 5A–5E, drugs were used to further elucidate the detailed mechanisms of mitochondrial respiration. In brief, the initial cellular OCR measures basal mitochondrial respiration. Oligomycin is a fungal product that inhibits eukaryotic ATP synthase, allowing quantification of the OCR for ATP synthesis [36]. FCCP is an ionophore, also known as an uncoupling agent, which causes proton leakages that accelerate the electron transfer system, allowing the spare respiratory capacity to be calculated [37]. Rotenone and Antimycin A shut down the electron transport chain, and the OCR difference between this treatment and FCCP allows the maximal respiration to be calculated [38]. The basal respiration, maximal respiration, and spare respiration capacity were lower in LPS-stimulated RAW 264.7 cells than in unstimulated control cells (Fig 5B, 5C and 5E). These observations are in accordance with the “Warburg Effect,” in which inflammatory cells perform glycolysis and lactate production, despite the abundance of oxygen [2]. The positive control, PO, successfully increased the OCR that had been reduced by LPS (Fig 5B, 5C and 5E). Of interest, the OCR was further upregulated in the experimental RBO-treated group, indicating that RBO enhances mitochondrial respiration and has subsequent anti-inflammatory properties.

While down-regulated inflammatory responses in LPS-driven M1 polarized macrophages were demonstrated in this study, it is noteworthy that homeostasis is required for macrophages at naïve and/or resolving stages [39]. In this regard, dietary supplement of RBO was previously examined for non-polarized M0 BMDM and anti-inflammatory M2 BMDM and results exhibited no effect on both M0 and M2 cells [40], indicating that RBO does not disrupt any homeostasis for infectious or inflammatory insults.

## Conclusion

Growing evidences indicate that dietary components regulate inflammation but their detailed mechanisms are poorly known. In the current study, the role of rice bran oil in macrophage functioning, which cues a series of inflammatory responses, was studied in murine models. Dietary lipids are a major source for cellular energy production through β-oxidation and subsequent mitochondrial respiration. Thus, the changes in mitochondrial respiration, cytokine production, and expression of activation markers following rice bran oil intervention were assessed both *in vitro* and *in vivo*. The results indicate that rice bran oil enhances the mitochondrial respiration and down-regulates inflammatory responses in murine macrophages. The current study, for the first time, revealed the contribution of dietary rice bran oil in alteration of cellular energy metabolism. The apparent limitation of the current study, however, lies in the fact that only fatty acids are considered for the beneficial effects of RBO. Indeed, RBO contains more than lipids, including but not limited to γ-oryzanol and tocopherols [18]. In addition, there is no mechanism proposed by which the balance between glycolysis and oxidative phosphorylation regulates the inflammatory function of macrophages, even though a pile of studies indicate the two are closely associated [1,41,42]. Dissected studies, as well as clinical investigations for validation of animal results, in the future are required for their detailed contributions and interactions on anti-inflammatory effects.
